# Young-onset versus late-onset colorectal cancer: clinicopathological features and survival outcome: a decade-long analysis from a middle Eastern tertiary center

**DOI:** 10.1186/s12957-025-04010-x

**Published:** 2025-10-09

**Authors:** Ammar Aleter, Ali Toffaha, Ejaz Ahmed Latif, Mahwish Khawar, Ibrahim Amer, Samer A. Hasan, Mahmood AL-Dhaheri, Ayman Ahmed, Ayman El-Menyar, Mohamed Abu Nada, Amjad Parvaiz

**Affiliations:** 1 Colorectal Surgery Unit, Hamad Medical Corporation, Doha, Qatar; 2 Clinical Research, Trauma & Vascular Surgery, Hamad Medical Corporation, Doha, Qatar; 3 Clinical Medicine, Weill Cornell Medical College, Doha, Qatar; 4 Champalimaud Foundation, Lisbon, Portugal; 5 Trauma & Vascular Surgery Section, Hamad Medical Corporation & Weill Cornell Medical College, Doha, Qatar

**Keywords:** Young, Colorectal cancer, Late-onset, Clinicopathological features, Survival analysis, Recurrence, Post-curative treatment

## Abstract

**Background:**

Colorectal cancer (CRC) incidence is reported to be declining overall in many countries but growing among young adults. The updated American Cancer Society (ACS) guidelines recommend screening starting at age 45 years. We sought to evaluate population-level trends in young colorectal cancer (yCRC) epidemiology in Qatar, a country in the Middle East.

**Methods:**

Between January 2010 and December 2020, we included 1529 patients from the National Registry Database with a 5-year follow-up. The patients were divided into two groups. Group 1 (*n* = 380, ≤ 45 years) and group 2 (*n* = 1149, > 45). The epidemiological and clinicopathological features were analyzed and compared in the two groups.

**Results:**

The annual incidence rate of CRC in Qatar from 2010 to 2020 ranged from 5.3 to 7.2 per 100,000 population, with an average of 5.79 per 100,000 population over this period. The overall prevalence among males was 65.6%. The incidence of CRC in young patients was approximately 1 in every four patients (24.8%). Males comprised almost two-thirds of the entire CRC cohort, yCRC, as well as the old-onset CRC cohort. The poorly differentiated CRC (including mucinous and signet ring features) was more prevalent in group 1 compared to group 2 (21.2% vs. 8%) (*p* = 0.001). Advanced CRC stages (III, IV) were significantly higher among the yCRC patients, with 63.3% of patients in group 1 diagnosed with advanced stages, compared to 59.6% (*p* = 0.001). Young patients with CRC were found to have more rectal involvement, with 35.6% of patients ≤ 45 years old compared to 23.9% in patients > 45 years old (*p* = 0.001).

**Conclusion:**

The reported incidence rate is approximately one-quarter of the newly diagnosed patients in Qatar. Patients with yCRC have a more aggressive and poorly differentiated histological type. The incidence of rectal cancer is higher in younger patients. Public awareness and screening policy have been implemented for better management.

## Highlights

• The increasing incidence rate of colorectal cancer in the young population is becoming a worrisome fact, according to multiple studies around the globe.

• There are still controversies regarding the clinicopathological features of the disease in younger patients and survival post-treatment.

• Although the younger patients in this study had more advanced diseases with inferior histological differentiation, the percentage of treatments with curative intent was similar in the young and old groups.

• The survival outcomes over five years were also comparable for both young and older patients.

• The recommended age for screening for colorectal cancer should be revisited worldwide.

• Treatment with curative intent should be initiated more frequently in younger patients, even in those with locally advanced disease or poor histological differentiation.

## Introduction

Colorectal cancer has historically been considered a disease of older adults. However, a concerning trend of increasing CRC incidence in younger populations, particularly those under 50 years old, has emerged [1–3]. Multiple studies are reporting an increase in the prevalence of colorectal cancer among young patients. This shift has prompted changes in screening guidelines, such as the American Cancer Society’s recommendation to begin screening at age 45 [4]. However, the epidemiological and clinicopathological features of CRC in young adults remain an evolving area of study. The “Screen for Life” program in Qatar targets average-risk adults at age 50–74 years using the fecal immunochemical testing (FIT). The screening service can be accessed by invitation from the dedicated cancer screening call center, physician referral, or self-referral [5]. In case the screening test is positive, the subject will be referred to undergo colonoscopy at the tertiary hospital. The average-risk adults include subjects without inflammatory bowel disease, familial adenomatous polyposis, hereditary nonpolyposis colorectal cancer, or positive family history of colorectal neoplasia [5, 6].

There are multiple established hereditary gene mutation found in yCRC such as familial adenomatous polyposis (APC), MUTYH-associated polyposis (MAP), juvenile polyposis syndrome, lynch syndrome and microsatellite chromosomal instability [7]. These genetic germline mutations are not common overall and comprise only 5% of all CRC cases and around 20% of patients with yCRC according to some studies [8, 9]. Lynch syndrome is the second most common cancer syndrome in Qatar after the hereditary breast and ovarian cancer [10]. The prevalence of Lynch syndrome among affected CRC patients and unaffected patients in Qatar is 22% and 2.2% respectively [11]. Notably, Qatar is small Middle Eastern Arab country in which the rate of consanguineous marriage is high, and the expatriates represents 80–85% of the population.

The existing literature presents conflicting data regarding the characteristics and outcomes of young-onset CRC [12]. While some research suggests that more aggressive tumors and decreased survival rates [3, 13] are associated with older adults, others have found no significant differences compared to younger adults [14, 15]. Another questionable feature of the early-onset CRC is the advanced stage. Conflicting data from multiple studies suggested an advanced stage of diagnosis for patients with young-onset CRC compared to those with late-onset CRC. In contrast, other studies could not demonstrate any variations [16]. This ambiguity underscores the need for further research specifically focused on young adult populations.

The survival outcome has been trending recently. Multiple studies suggested a worse outcome for patients with young-onset CRC [16–18], especially in advanced stages or if the tumor has an aggressive pathology. However, others could not find any difference in survival, and some even found a superior outcome for younger patients [19]– [20].

Data from the Middle East on young-onset colorectal cancer are scarce. Therefore, this study seeks to address the existing knowledge gap by investigating the characteristics and outcomes of young adults with CRC treated at our tertiary care center over a decade. By examining clinical and pathological features, as well as survival data within a specific population, we aim to contribute a more nuanced understanding of young-onset CRC. This information could have important implications for early detection strategies, tailored treatment approaches, and ultimately, improved outcomes for young adults facing this challenging diagnosis. This investigation is particularly crucial given the longer life expectancy of young-onset patients, which underscores the potential long-term impact of this disease [2].

## Methods

### Study design

This is a retrospective study with a follow-up. The study population comprised 1,529 patients retrieved from the National Cancer Registry Database (NCRD) of Qatar between January 2010 and January 2020. Data from five regional hospitals were consolidated in this comprehensive cancer registry, which employs robust mechanisms to ensure the security and validity of the registered data.

The diagnosis of colorectal cancer, investigations performed, treatment administered, and patient outcomes were all extracted from the NCRD. We conducted this study in compliance with the principles of the Declaration of Helsinki. The study’s protocol was reviewed and approved by the Institutional Review Board of Hamad Medical Corporation, Medical Research Center (MRC-01-24-498). The data was anonymized, retrospective, and non-interventional; thus, informed consent was waived.

The study population was divided into two cohorts based on age: those aged 45 years or younger (yCRC) and those older than 45 years. This age delineation aligns with the updated American Cancer Society guidelines, which recommend colorectal cancer screening beginning at age 45. Epidemiological and clinicopathological characteristics were compared between the two age groups to assess disease staging and aggressiveness. Colorectal cancer patients were further categorized by location into right-sided colon, left-sided colon, and rectum. Prospective follow-up was conducted over the subsequent five years, up to January 2025, to evaluate treatment modalities, recurrence rates, overall survival, and disease-free survival in relation to patient age and cancer stage.

### Statistical analysis

Data was presented as proportions, means, and 95% confidence intervals whenever applicable. Descriptive analysis included Fisher’s exact test or Pearson’s chi-squared test for categorical data, and the Kruskal-Wallis test for non-parametric distribution of mean data. Patients who died were censored according to their date of death. Patients alive at the last contact date had their survival time censored at the date of last contact. Survival means were presented with an associated 95% confidence interval (CI). The Kaplan-Meier curve was used to represent the 5-year overall survival and disease-free survival rates. A p-value of < 0.05 was considered statistically significant. All statistical analyses were performed using IBM Corp. (2022). IBM SPSS Statistics for Windows, Version 29.0. Armonk, NY: IBM Corp.

## Results

**Fig. 1:**
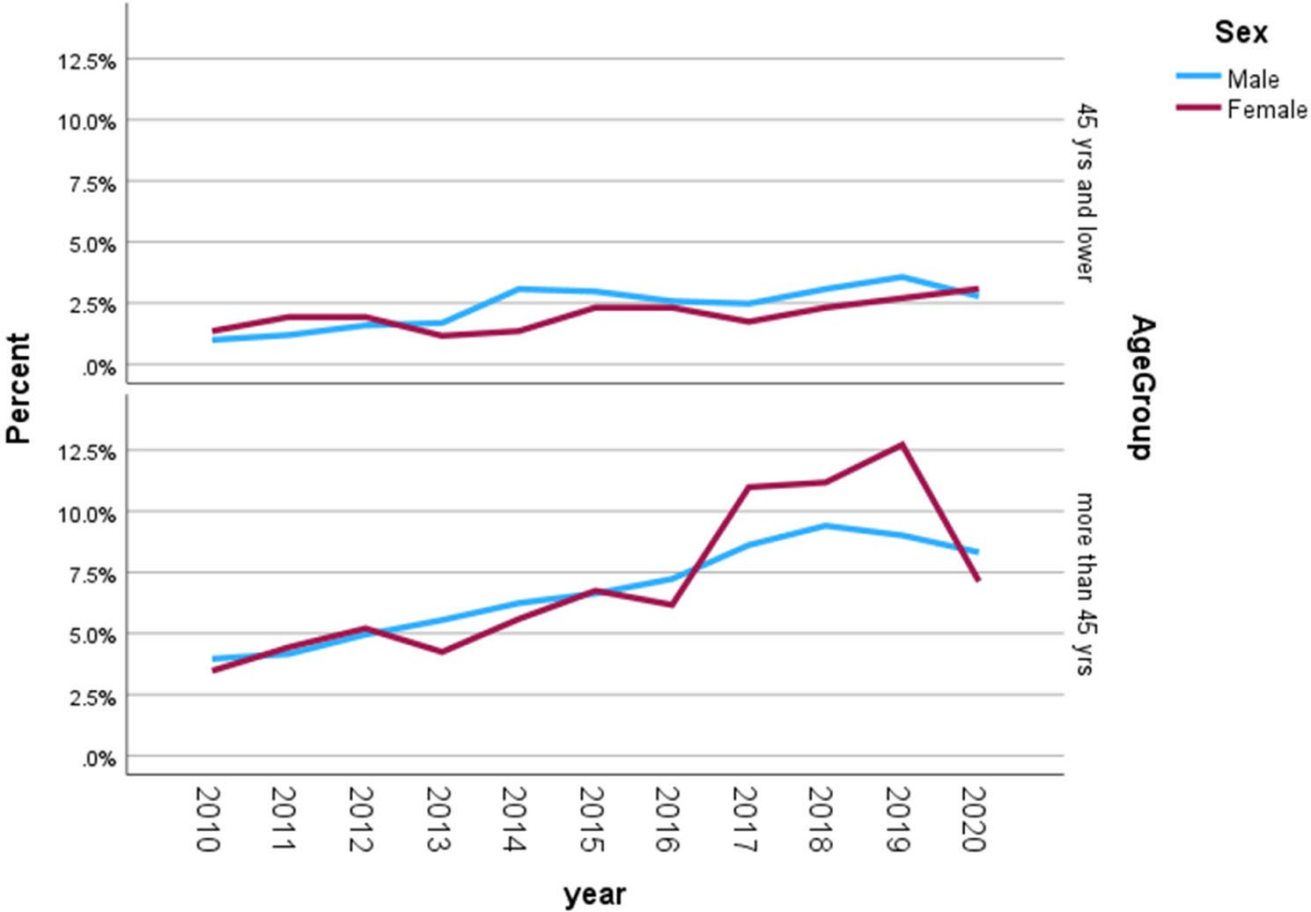
The incidence of colorectal cancer in Qatar per year, stratified by age and gender

During the study period, a total of 1,529 patients were diagnosed with colorectal cancer in Qatar. Of these, 380 (24.8%) patients were 45 years of age or younger, while 1,149 (75.2%) patients were older than 45 years. Males comprised almost two-thirds of the entire CRC cohort, yCRC, as well as the old-onset CRC cohort. The annual incidence rate of cancer in Qatar from 2010 to 2020 ranged from 5.3 to 7.2 per 100,000 population, with an average of 5.79 per 100,000 population over this period (adjusted for people aged 19 and above). Figure 1; Table 1 show the incidence of colorectal cancer in Qatar per year, stratified by age and gender. In patients aged 45 years and below, there was a slight steady increase in the CRC over the years in males and females. In patients older than 45 years, there was a significant increase over the years in both sexes; however, females showed a greater rise in CRC after 2016 compared to males. Overall, the proportion of cases increased from 5.0% in 2010 to 13.5% in 2019. In yCRC, the proportion of male cases increased from 3.8% in 2010 to 13.7% in 2019, while the proportion of females increased from 6.1 to 13.9%. In old-onset CRC, the proportion of male cases increased from 5.0% in 2010 to 12.2% in 2019 and from 4.5 to 16.3% in females.

**Table 1:** Incidence of colorectal cancer per year stratified by age and gender

	All CRC patients			Patients ≤ 45 years			Patients > 45 years		
Year	Overall %	Males %	Females %	Overall %	Males %	Females %	Overall %	Males %	Females %
2010	4.9	5.0	4.8	4.5	3.8	6.1	5.0	5.3	4.5
2011	5.7	5.3	6.4	5.8	4.6	8.7	5.6	5.6	5.7
2012	6.7	6.5	7.1	6.9	6.1	8.7	6.7	6.7	6.7
2013	6.6	7.2	5.4	6.1	6.5	5.2	6.8	7.5	5.4
2014	8.5	9.3	6.9	10.1	11.8	6.1	8.0	8.4	7.2
2015	9.4	9.6	9.1	11.1	11.5	10.4	8.9	9.0	8.7
2016	9.4	9.8	8.5	10.1	9.9	10.4	9.1	9.8	7.9
2017	11.6	11.1	12.7	9.0	9.5	7.8	12.5	11.6	14.1
2018	12.8	12.5	13.5	11.4	11.8	10.4	13.3	**12.7**	14.4
2019	**13.5**	**12.6**	**15.4**	**13.3**	**13.7**	12.2	**13.6**	12.2	**16.3**
2020	10.8	11.1	10.2	11.7	10.7	**13.9**	10.5	11.2	9.2
Total	1529 (**100%**)	1010 (**66%**)	519 (**34%**)	377 (**24.6%)**	262 (**69.5**%)	115 (**30.5**%)	1152 (**75.4%)**	748 (**65**%)	404 (**35**%)

The data indicated that the percentage of female patients with colorectal cancer was higher in the older age group, although this difference was not statistically significant (*p* = 0.22). In both age groups, the percentage of CRC patients was higher among males. (Table 1) The majority of CRC cases were observed among individuals from the Middle East and North Africa region, with a higher probability in the older age group. Conversely, patients from the South Asia and Southeast Asia regions were more likely to have young-onset CRC (*p* = 0.001). Patients from the Europe and North America regions were more likely to be in the older (> 45 years) age group (*p* = 0.02). Table 2 shows a comparative analysis of the demographic characteristics between the two age groups.

**Table 2:** Comparative analysis of demographic characteristics

Category	Young CRC	Older CRC	*p*-value
Number of patients	380 (24.8%)	1149 (75.2%)	--
Gender			
Male Female	264 (69.5%) 116 (30.5%)	740 (64.4%) 409 (35.6%)	0.22
Geographical region			
Middle East and North Africa	(145) (38.2%)	(770) (67.1%)	0.001
South Asia (India, Pakistan, Sri Lanka, Bangladesh, etc.)	(128) (33.7%)	(203) (17.6%)	0.001
Southeast Asia (Philippines, Indonesia, Thailand, etc.)	(77) (20.3%)	(82) (7.1%)	0.001
Europe and North America	(13) (3.4%)	(75) (6.5%)	0.02
Others	(17/380) (4.4%)	(19/1149) (1.7%)	0.15

### Anatomical location and histopathological grade

Compared to older CRC patients, yCRC patients were more frequently diagnosed with rectal cancer (35.6% vs. 23.9% for older patients, *p* = 0.001). In contrast, older patients were more likely to present with left-sided colon cancer (*p* = 0.001). No significant difference was observed in the incidence of right-sided colon cancer between the two age groups (*p* = 0.90). Additionally, yCRC patients exhibited a higher proportion of poorly differentiated tumors compared to their older counterparts (21.2% vs. 8%, *p* = 0.001).

### Cancer staging and therapeutic management

The analysis revealed no statistically significant difference in the proportion of patients diagnosed with early-stage (stage I/II) colorectal cancer between the younger and older age groups (*p* = 0.20). However, a higher percentage of young-onset colorectal cancer patients were found to have advanced-stage (stage III/IV) disease compared to older patients (63.3% vs. 59.6%, *p* = 0.001).

Out of the 1,529 patients diagnosed with colorectal cancer, 1,071 (70%) underwent curative surgical resection with or without chemotherapy. Most patients with stage I and II disease, regardless of age, underwent curative resection. For those with stage III and IV disease, 54.2% of yCRC patients underwent curative surgical resection compared to 57.7% of older patients (*p* = 0.31). Despite the higher proportion of yCRC patients presenting with advanced-stage disease, this did not significantly impact the likelihood of receiving curative surgical treatment, as shown in Table 3.

**Table 3:** Comparative analysis of locations of tumors, histological grades, and the cancer staging by age

	Young colorectal cancer	Older colorectal cancer	*p*-value
**Cancer Location** Right-sided colon cancer Left-sided colon cancer Rectal cancer **Grade**	88/360 (24.4%) 144/360 (40%) 128/360 (35.6%)	277/1143 (24.2%) 593/1143 (51.9%) 273/1143 (23.9%)	0.90 0.001 0.001
Well differentiated	84/359 (23.4%)	322/1005 (32.0%)	0.001
Moderately differentiated	199/359 (55.4%)	602/1005 (60.0%)	0.14
Poorly differentiated (undifferentiated/signet ring cell/poor differentiation with mucinous features)	76/359 (21.2%)	81/1005 (8.0%)	0.001
**Disease stage**			
Stage I/II	138/376 (36.7%)	458/1134 (40.4%)	0.20
Stage III/IV (advanced) **Curative resection according to stage** Stage I/II Stage III/IV	238/376 (63.3%) 129/138 (93.5%) 129/239 (54.2%)	676/1134 (59.6%) 422/457 (92.3%) 391/677 (57.7%)	0.001 0.96 0.31

### Survival outcomes

**Fig. 2:**
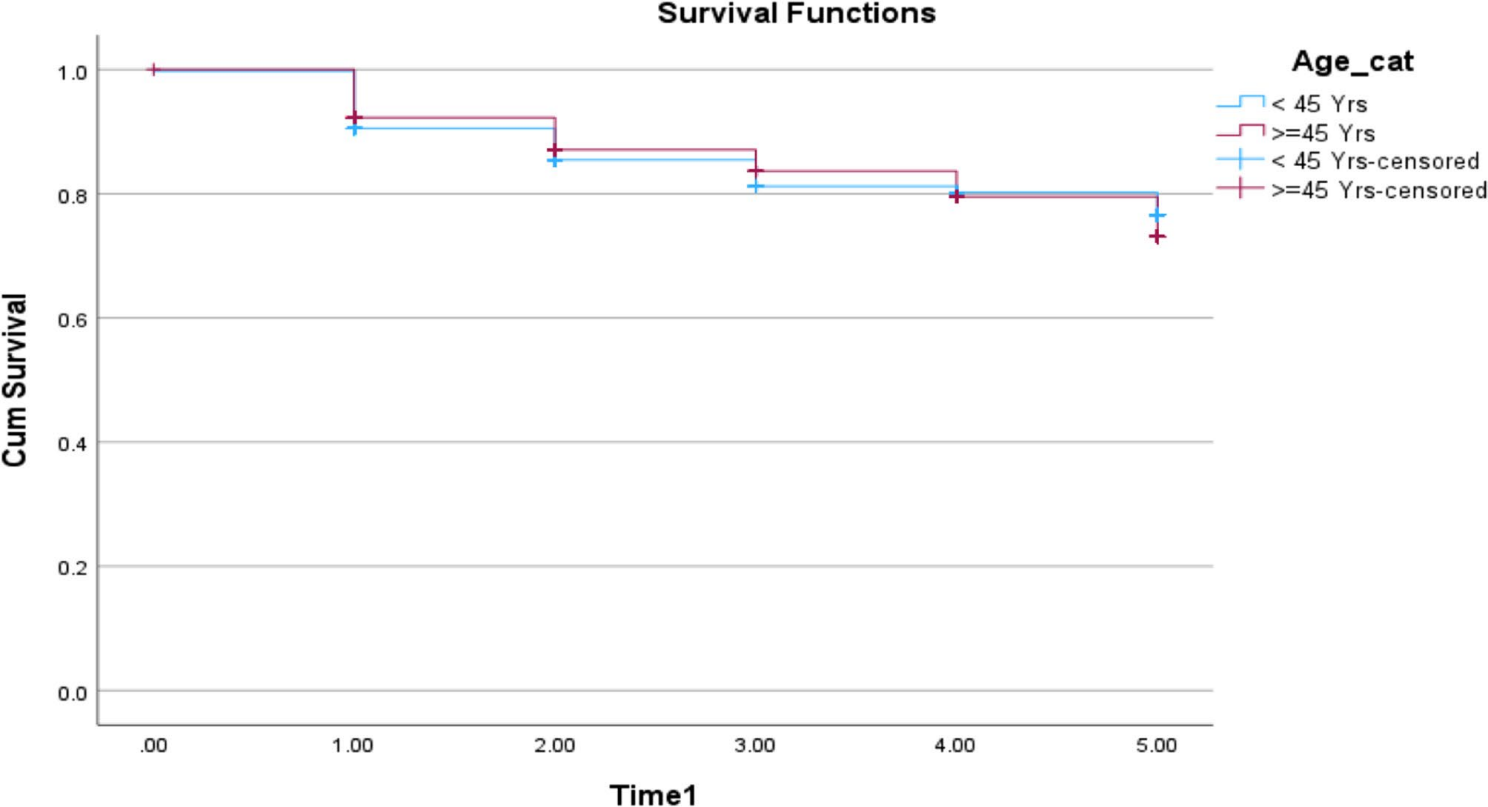
Kaplan-Meier curve illustrates the difference in overall survival over 5 years between the two age groups. Log rank (Mantel-Cox); P=0.758

The patients were followed up for at least 5 years after diagnosis, and their relative survival rates were calculated to determine overall and disease-free survival. When comparing 5-year overall survival (OS) rates between the yCRC and older patient groups, no statistically significant difference was found (73.1% for yCRC and 76.6% for older patients, *p* = 0.758) (Fig. 2 shows the Kaplan-Meier curve for overall survival).

For patients who underwent curative surgical resection following diagnosis, the 5-year disease-free survival (DFS) rates were not significantly different when stratified by age (78.3% for patients with yCRC and 81.9% for those with older patients, *p* = 0.168).

**Fig. 3:**
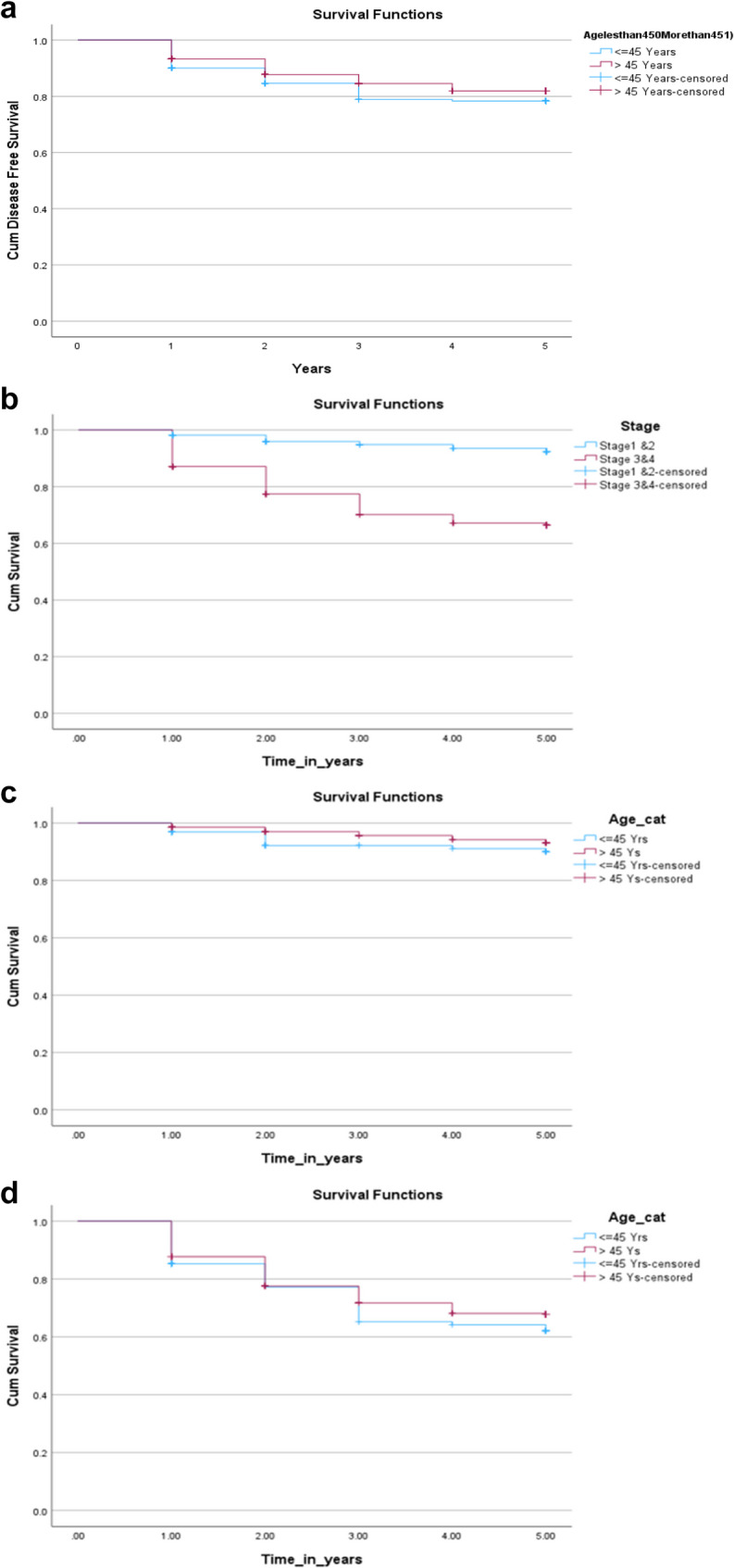
**a** Kaplan-Meier curve illustrates the difference in disease-free survival over 5 years between the two age groups: Log rank (Mantel-Cox); P=0.164. **b** Kaplan-Meier curve showing disease-free survival (DFS) post curative resection over 5 years stratified by stage (stage I/II CRC compared to stage III/IV) (Log rank (Mantel-Cox); p<0.001). **c **Kaplan-Meier curve showing disease-free survival (DFS) over 5 years for stage I/II CRC (Log rank (Mantel-Cox); p<0.212)

The study results are presented visually in Fig. 3 **a**, **b** and **c** which displays the Kaplan-Meier curve for disease-free survival. Table 4 provides a quantitative comparison of OS and DFS percentages and means between the younger-onset colorectal cancer and older patient groups.

**Table 4:** Comparative analysis of the overall survival and Disease-free survival of CRC

	Young colorectal cancer	Older colorectal cancer	*p*-value
Percentage of overall survival (5 years)	73.1%	76.6%	0.758
Mean of overall survival (years)	4.37 (4.22–4.51)	4.425 (4.34–4.50)	
Percentage of Disease-free survival (5 years)	78.3%	81.9%	0.164
Mean of Disease-free survival (years)	4.319 (4.14–4.49)	4.475 (4.38–4.56)	

Stage-stratified survival analysis demonstrated that patients with early-stage (I/II) colorectal cancer had a 5-year disease-free survival rate of 92.4%, significantly higher than the 66.4% rate for those with advanced-stage (III/IV) disease (*p* = 0.001). When examining disease-free survival based on age, no statistically significant difference was found between younger-onset colorectal cancer patients and older-onset patients for either early-stage (90.0% vs. 93.1%, *p* = 0.21) or advanced-stage disease (*p* = 0.29). Table 5 presents a comparative analysis of disease-free survival percentages and means for the two age groups across various stages of cancer.

**Table 5:** Comparative analysis of disease-free survival percentages and means by age across different cancer stages.

	Young colorectal cancer (≤ 45-year-old) *n* (%) (95%CI)	Older colorectal cancer (> 45 years old) *n* (%) (95%CI)	*p*-value
Percentage of Disease-free survival (5 years) for stage I/II	90.0%	93.1%	0.212
Mean of Disease-free survival (years)	4.724 (4.54–4.90)	4.855 (4.79–4.91)	
Percentage of Disease-free survival (5 years) for stage III/IV	62.1%	67.8%	0.292
Mean of Disease-free survival (years)	3.919 (3.63–4.20)	4.051 (3.89–4.20)	

## Discussion

This epidemiological and clinicopathological study is one of the first studies, to our knowledge, that assesses young patients diagnosed with colorectal cancer with 5 years of follow-up in the Arab Middle East. In Qatar, the current population is 3,100,00 according to the Worldometer’s elaboration of the latest United Nations data [21] and based on the present analysis, the estimated CRC in 2025 will be 8.23 per 100,000 population and age-adjusted estimates (≥ 19 years) of 9.95 per 100,000 population. The present study, using data from 2010 to 2020 revealed an average CRC annual incidence of 7.5/100,000/year. Consistent with most previous reports, there is a rise in the proportion of colorectal cancer cases among younger individuals compared to older patients [22]. While some studies have reported conflicting findings, most of the literature supports the observed trend of an increase in the proportion of colorectal cancer cases among younger individuals [15]. The recent preponderance of data, primarily from North America, Asia, and Europe, has already established awareness of this emerging and concerning trend [23]– [24]. A recent bibliometric analysis has revealed several gaps in research on CRC screening in Arab countries [25]. This gap could potentially be attributed to lack of a comprehensive screening strategy in the region due to low resources. On the other hand, Qatar has implemented an organized population-based screening program [25]. A large epidemiological study of CRC in Saudi Arabia, showed that there was a markedly increasing incidence of CRC from 2006 to 2016 due to the large-scale screening program [26]– [27].

Prior study from our institution between 1984 and 1998 showed 24 CRC cases per year [28], while the present study reported around 139 cases per year, this discrepancy is mainly due to lack of screening and diagnostic tools in the past century [28, 29]. Our study found the percentage of young-onset colorectal cancer patients over the study period to be remarkably elevated (24.8%), even exceeding the yCRC incidence rates globally, observed in countries such as Australia (16.5%), Puerto Rico; 15.2%, New Zealand (14.8%) and South Korea (14.3%) [24, 30]. This may be partially attributable to the substantial proportion of young expatriate workers from South and Southeast Asia residing in Qatar, as well as the generally younger overall population demographics compared to those in European nations (the Median age in Qatar is 33.5 years) [31]. The high incidence of yCRC in Qatar could reflect a combination of risk factors, including genetic predisposition, environmental exposures, and lifestyle habits among the diverse population in the region [32]. Increased consumption of processed meat along with low fiber, low vegetables and fruit diet could be dietary risk that along with lack of awareness of CRC symptoms could lead to increase the CRC in young patients [33]. A prior study in 2020 from the region showed that the awareness of CRC symptoms and risk factors was low among the at-risk population [34]. Additionally, limited access to early detection and preventive healthcare services for specific demographic groups may contribute to the disproportionate burden of yCRC. Further research is needed to elucidate the underlying drivers of this concerning trend and inform the development of targeted interventions to address this emerging public health challenge. Therefore, the improvement of the diagnostic tools, awareness program, and establishment of the National Center for Cancer Care and Research (NCCCR), which was launched in 2011 (the premier cancer hospital for the State of Qatar) will be of high importance for better registration and management.

The data indicate that colorectal cancer in young patients is more commonly located in the left-sided colon and rectum, which aligns with findings from multiple studies conducted in the United States, Asia, and Australia [35–38]. However, an extensive population-based study from the United Kingdom reported a higher incidence of right-sided colon cancer in young-onset colorectal cancer patients [15]. These divergent results underscore the importance of performing a comprehensive colonoscopy when young adults present with concerning symptoms, to ensure thorough evaluation of the entire colorectal region.

The higher proportion of young-onset colorectal cancer patients presenting with advanced-stage disease compared to older patients has been consistently reported in the literature [17, 39–41]. These reports could be attributed to a combination of factors, including delayed diagnosis due to the atypical presentation of colorectal cancer in young adults, as well as potentially more aggressive tumor biology [40]. These findings were not matched in other recent papers, suggesting no increase in advanced stage for patients with yCRC [19, 42].

Studies have suggested that yCRC may exhibit more aggressive histological subtypes and a higher propensity for lymph node involvement and distant metastases, leading to a greater proportion of patients being diagnosed at later stages [13, 43]. Additionally, the nonspecific symptoms and lower clinical suspicion of colorectal cancer in younger individuals may contribute to delayed diagnoses, further exacerbating the higher incidence of advanced-stage disease in this population [17, 40].

The existing literature, including data from the Surveillance Epidemiology and End Results database, has suggested that yCRC is more aggressive, with patients more likely to present with advanced-stage disease compared to older patients [38, 40]. Our findings corroborate these previous reports. Specifically, 21.8% of yCRC patients exhibited an aggressive histological subtype, significantly higher than the 8% observed among patients older than 45 years. Furthermore, a greater proportion of yCRC patients were diagnosed with stage III or IV disease compared to 59.6% in the older age group.

Despite the higher proportion of yCRC patients presenting with advanced-stage disease, the likelihood of receiving curative surgical treatment in our study was comparable between the younger and older age groups. Moreover, the survival analysis, including overall survival, disease-free survival, and stage-stratified survival, did not reveal any statistically significant differences between the two age cohorts. These findings suggest that even with a more aggressive histopathological pattern and a greater percentage of yCRC patients diagnosed at advanced stages, the probability of curative resection and post-diagnosis survival in yCRC patients remains comparable to that of older patients. These results are consistent with multiple previous studies conducted in Asia, Europe, and the United States, which have reported equivalent or even superior survival outcomes for yCRC patients [36, 44–47]. The comparable survival outcomes observed in our study may be attributable to the younger patients’ potentially better tolerance to aggressive treatment regimens and their longer life expectancy, which may offset the impact of more advanced diseases at presentation.

Young CRC patients with distant metastasis on initial diagnosis accounted for (43.2%) of patients with advanced stage (stage III/IV), while as for older patients, the percentage was close (40.2%) in the present study. Curative treatment could be offered for patients with distant metastasis, especially with oligo metastasis and no extensive peritoneal disease [48]. Decisions should be made through a multidisciplinary committee to tailor the treatment individually [48]. For chemotherapy naïve patients with unresectable metastatic CRC, Neoadjuvant chemotherapy should be offered as first-line therapy and Pembrolizumab should be offered to patients with microsatellite instability-high or deficient mismatch repair metastatic CRC [49]. As for colon cancer, Neoadjuvant treatment is used mainly with oligo metastasis or in case of locally advanced disease [50].

Neoadjuvant treatment for patients with locally advanced cancer was found to downstage the tumor, achieve higher percentage of negative resection margin and decrease the residual or recurrence disease within 2 years according to recent randomized control trial [50]. Total neoadjuvant therapy (TNT) has recently become the cornerstone of managing locally advanced rectal cancer with an involved circumferential resection margin (CRM), T4 disease, lateral pelvic node involvement or extramural vascular invasion (EMVI) [51]. Multiple regimens were described for TNT that differ significantly in terms of radiation dosage, chemotherapy regimen and order of treatments administered [51]. Upfront surgery is suggested if no indication for neoadjuvant treatment was found [51].

Regarding molecular analysis, KRAS and BRAF genes evaluation is being done if patients need neoadjuvant/adjuvant chemotherapy treatment for consideration of epidermal growth factor receptor (EGFR) therapy [52]. Microsatellite chromosomal instability (MSI) is being done for most of our patients. Mainly, if they are below age of 50 or lynch syndrome is suspected. However, full molecular genetic testing is costly, and it is done only if there is high suspicion of hereditary genetic mutation.

Table 6 summarizes studies published over the past 5 years that discussed the clinicopathological features and survival analysis of the disease [17, 18, 36, 40–42, 53–55]. The observed trends in clinicopathological features and survival outcomes for yCRC patients highlight the importance of early detection and screening, as well as a heightened level of clinical suspicion for colorectal cancer, even in younger individuals presenting with nonspecific gastrointestinal symptoms. Given the potentially more aggressive tumor biology in yCRC, it is crucial to develop dedicated screening strategies and optimize treatment approaches to improve outcomes for this high-risk population. The data presented here provide valuable insights to inform the development of evidence-based guidelines and clinical practice recommendations for the management of young-onset colorectal cancer.

**Table 6:** Summary of the most recent studies discussing the clinicopathological features and prognosis of colorectal cancer in young patients.

Author	Country	Patient Number (early onset/late onset) (%)	Gender prevalence (%)	Mean age	Most common tumor location (%)	Percentage of poorly differentiated histopathology (early/late onset) (%)	Advanced stage at diagnosis (III/IV) (early/late onset) (%)	Treatment/intervention received (early/late onset) (%)	Survival/outcome (3–5 years)
Gusman V et al. (2020) [40]	USA	(269/2802) (9.6%)	Male (54%)	43	Left-sided (41%)	(21/17) (*P* = 0.53)	(77/62) (*P* = 0.01)	-	-
Surek A et al. (2021) [47]	Turkey	(56/267) (21%)	Male (65%)	42.2	Rectum (42.8%)	(25/12.32) (*P* = 0.018)	(46.42/33.17) (*P* = 0.066)	-	Distant metastasis: no difference. Local recurrence: higher for early onset (3.5% vs. 2.36%) (*P* = 0.729)
Cercek A et al. (2021) [36]	USA	(759/1446) (52.4%)	Male (53.2%)	-	Left-sided	No significant difference	No significant difference.	Chemotherapy treatment (neoadjuvant or adjuvant): no significant difference	Radiographic response to chemotherapy: no significant difference. (*P* = 0.7) No significant difference in overall survival
Park KS et al. (2022) [39]	Korea	(111/1015) (9.8%)	Male (60.4%)	-	Left-sided (41.4%)	(10.8/6.7) (*P* = 0.003)	(51.3/44) (*P* = 0.394)	-	5-year overall survival: (86.9/78.6) (*P* = 0.229) 5-year disease-free survival: (74.0/69.3) (*P* = 0.517)
McClelland PH et al. (2022) [17]	USA	(35084/205688) (14.5%)	Male (53.8%)	-	Left-sided (Rectosigmoid and descending colon) (41.3%)	-	- Early-onset was associated with advanced tumor stage. - Stage III: (34.2/27.9) (*P* < 0.001) Stage IV: (27.1/20.7) (*P* < 0.001)	-	− 5-year disease-specific survival (66.4/68.2) (*P* = 0.031). - Male sex, older age, advanced stage, rectal and/or cecal primary, and earlier year of diagnosis were independently associated with increased mortality.
Park SB et al. (2023) [53]	Korea	(47/134) (25.9%)	Male (53.2%)	44.9	Distal location (left-sided and rectum) (70.2%)	(6.4/15.7) (*P* = 0.14)	Advanced stage (LN metastasis) (48.9/50.7) (*P* = 0.59)	-	- Overall, the cumulative survival was lower in late-onset CRC patients with vascular invasion. (*P* = 0.05). - Other variables (Age, sex, TNM stage, BMI) did not affect survival.
Liu H et al. (2024) [54]	China	(991/3581) (21.6%)	Male (54.9%)	-	Rectum (46.5%)	-	- Stage III: (46.4/44.2) Stage IV: (36.7/34.8) No significant difference. - Early onset has a higher percentage of widespread metastasis (19.7/14.1) (*P* < 0.001)	Curative resection (84.4/83.6) (*P* = 0.612) Adjuvant chemotherapy, targeted therapy, and radiotherapy were more common in early onset (*P* < 0.001)	-
Liao CK et al. (2024) [55]	Taiwan	(1240/4464) (21.7%)	Male (51.1%)	44	Rectum (39.4%)	(15.1/9.4) (*P* < 0.001)	Stage III: (62.4/50.3) (*P* < 0.001)	Curative resection (80.2/87) (*P* < 0.001) Adjuvant therapy (57.4/46.4) (*P* < 0.001) Combined Surgery (34.1/27.6) (*P* < 0.001)	Cancer-specific survival (stage I/III): no difference between the two groups. (Stage IV): worse in early onset (*P* = 0.012)
Khan MS et al. (2025) [18]	Pakistan	(78/84) (48.1%)	Male (50%)	-	Rectum (71.7%)	(44.8/11.9) (*P* = 0.013)	Stage III: (71.7%/53.5%) (*P* = 0.031)	Complete response for neoadjuvant therapy for rectal cancer only (11.5/28.5) (*P* = 0.093)	2-year overall survival: (77.6/92.9). 2-year disease-free survival: (76.8/93.9) Distant recurrence (20.5/4.7) (*P* = 0.042)
Ionescu VA et al. (2025) [41]	Romania	(23/181) (12.7%)	Male (54.4%)	44.4	Left-sided (47.8%)	(39.1/14.2) (*P* = 0.010)	- Stage IIIB is the most common in both groups. - Stage IV is more frequent in early onset (P = NS).	-	-

The key areas that could be used to detect CRC in the young population include public awareness and screening campaigns focusing on the early symptoms and avoidable risk factors [34]. Decreasing the screening age to follow the international guideline from 50 to 45 could facilitate in diagnosing yCRC at earlier stages. Also, enabling an earlier and approachable screening tests such as fecal occult blood test and FIT test if there are red flag symptoms could lead to an earlier detection and treatment.

Limitations of this study include its single-center and retrospective nature, which may introduce potential biases, and the relatively small sample size, particularly in the younger-onset colorectal cancer group, which may limit the statistical power to detect subtle differences. Future prospective multicenter studies with larger cohorts are warranted to elucidate further the distinct clinicopathological features and optimal management approaches for young-onset colorectal cancer patients in the region.

## Data Availability

all data are given in the manuscript. Further information can be requested from the PI after a reasonable request and approval from the medical research center at HMC, Doha.
